# Does Hospital Competition Save Lives? Evidence from the English NHS Patient Choice Reforms*

**DOI:** 10.1111/j.1468-0297.2011.02449.x

**Published:** 2011-07-21

**Authors:** Zack Cooper, Stephen Gibbons, Simon Jones, Alistair McGuire

**Affiliations:** 1 London School of Economics

## Abstract

Recent substantive reforms to the English National Health Service expanded patient choice and encouraged hospitals to compete within a market with fixed prices. This study investigates whether these reforms led to improvements in hospital quality. We use a difference-in-difference-style estimator to test whether hospital quality (measured using mortality from acute myocardial infarction) improved more quickly in more competitive markets after these reforms came into force in 2006. We find that after the reforms were implemented, mortality fell (i.e. quality improved) for patients living in more competitive markets. Our results suggest that hospital competition can lead to improvements in hospital quality.

Across the developed world, health care spending accounts for a large and growing share of most countries’ gross domestic product (GDP).^1^ In an effort to slow the rate of spending growth and improve health system performance, a number of countries have enacted market-based health care reforms that have centred on increasing user choice and promoting competition between health care providers.^2^ These reforms have been primarily designed to create financial incentives in a sector that has typically been more state-directed and centrally controlled than others. However, there is not a consensus on how health care markets should be structured and the evidence on the impact of choice and competition on clinical quality is inconclusive (Dranove and Satterthwaite, 1992, 2000; Gaynor and Haas-Wilson, 1999; Sage *et al*., 2003; Gaynor, 2004). This article evaluates one recent set of market-based health reforms introduced in the English National Health Service (NHS) from 2002 to 2008, which focused on introducing patient choice and provider competition. We take advantage of the explicit introduction of choice and competition into the NHS in 2006 to create a quasi-natural experiment where we can examine whether greater exposure to competition prompted hospitals to improve their performance.

The recent English NHS reforms had three central elements (Department of Health, 2003). First, patients were given the ability to select the hospital they attend for surgery and the government provided publicly assessable information on provider quality to inform patients’ choices. Second, the government liberalised the hospital sector in England by giving publicly owned hospitals additional fiscal and managerial autonomy and encouraging private sector providers to enter the market and deliver care to publicly funded patients. Third, the government introduced a new case-based hospital reimbursement system that paid providers a fixed, centrally determined price for every procedure that they carried out. In sum, policy makers in the NHS hoped that their efforts to encourage patient choice would create quality competition between hospitals in England, which would prompt providers to improve their clinical performance (Department of Health, 2004).

In order to assess the impact of the recent NHS market-based reforms, we exploit the fact that the choice-based reforms will create sharper financial incentives for hospitals in markets where choice is geographically feasible. Specifically, we use a difference-in-difference (DiD) style estimator to test whether patient outcomes in more potentially competitive markets have improved at a significantly faster rate post-reform than in less competitive markets after all patients in England were formally given the ability to select their hospital in 2006. We measure these improvements in quality by examining changes in 30-day mortality rates for patients diagnosed with an acute myocardial infarction (AMI). Thirty-day AMI mortality is an appealing quality indicator because AMIs are easily clinically identifiable, AMI mortality is not subject to gaming or manipulation like many elective outcomes, and for patients with an AMI, there is a clear link between appropriate treatment and good outcomes (Bradley *et al*., 2006; Jha *et al*., 2007). Indeed, 30-day AMI mortality is frequently used in the literature assessing the relationship between competition and overall hospital quality.^3^

This work adds to the existing literature examining the impact of public sector reform on service quality. To evaluate the reforms, we use a DiD estimator and (*a*) develop a range of concentration measures and illustrate that our results are robust across each; (*b*) calculate concentration using elective patient flows and measure quality using outcomes for an emergency procedure (AMI), which mitigates the selection bias inherent in using quality measures based on the outcomes of elective procedures; (*c*) develop an instrument for market competition that exploits the variability in distance between a patient’s GP and their nearest four hospitals (which is largely a historical artefact) as an exogenous source of variation in the underlying market structure; and (*d*) present various tests of robustness that indicate that our estimates arise post-2005 are consistent across various alternative specifications of our estimator and are driven by hospital market structure and not by spurious associations with urban density.

Ultimately, we find that after the introduction of these reforms in 2006, our marker for service quality (AMI mortality) improved more quickly for patients living in more competitive hospital markets. Compared to the mean, AMI mortality has fallen approximately 0.31 percentage points per year faster in places that were one standard deviation higher on our market structure index (on a base mortality of 13.82% during the 2002–8 period). As a result we conclude that hospital competition within a market with fixed prices can improve patient outcomes.

This article is structured as follows. Section 1 examines the existing literature on the impact of hospital competition on quality. Section 2 outlines the recent NHS market-based reforms and presents our estimation strategy. Section 3 outlines our data, marker for service quality and the various measures of competition to define the ‘treated’ groups in our DiD estimations. Section 4 presents our results. Section 5 contains our conclusions.

## 1. Evidence on the Relationship Between Hospital Competition and Hospital Quality

There is a growing US literature analysing the impact of hospital competition on hospital quality and efficiency; however, the evidence for England is only just emerging (Propper *et al*., 2004,2008; Cooper *et al*., 2010a,2010b; Gaynor *et al*., 2010). Comprehensive reviews of this literature can be found in Gaynor (2004), Romano and Mutter (2004), Propper *et al*. (2006), Vogt and Town (2006), Cooper *et al*. (2010*b*)2010b.

A key trend emerging from this literature is that greater competition in markets with fixed prices generally leads to improvements in hospital performance (Gaynor, 2004,2006). Examining competition in a fixed price market in the US, Kessler and McClellan (2000) looked at the impact of hospital competition on AMI mortality for Medicare beneficiaries from 1985 to 1994. They find that in the 1980s, the impact of competition was ambiguous but, in the 1990s, higher competition led to lower mortality. Related work by Kessler and Geppert (2005) also found that competition reduced AMI mortality and that it also led to more intensive treatment for sicker patients and less intensive treatment for healthier patients. However, Gowrisankaran and Town (2003) found that increased competition in a fixed price market led to an increase in mortality, but argue that their results stem from the fact that hospitals in California were underpaid for treating Medicare patients with AMI. This hypothesis is consistent with research, which found that lower Medicare reimbursement rates led to increases in mortality, particularly in competitive markets (Shen, 2003).^4^

Nearly, all of the English literature on hospital competition examines an earlier set of NHS reforms – the 1990s internal market. This market allowed hospitals to compete on quality and price for bulk purchasing contracts but, in general, there is a near uniform consensus that the internal market never created significant financial incentives for hospitals to change their behaviour (Le Grand *et al*.^1998^; Klein, 1999; Le Grand, 1999). There is some evidence that prices fell during the internal market (Propper, 1996; Soderlund *et al*., 1997; Propper *et al*., 1998); however, Soderlund *et al*. (1997) suggest that higher competition was not associated with lower prices. Propper *et al*. (2004,2008) examined the impact of competition on clinical performance during this period. Both studies find that competition (measured using counts of hospitals within markets defined by 30 minute isochrones) was associated with lower hospital quality, as measured by AMI mortality, possibly because hospitals shifted resources towards reducing waiting times and improving other easily observed measures of performance.

More recently, several working articles, including Bloom *et al*. (2010), Cooper *et al*. (2010 a,2010 b), and Gaynor *et al*. (2010) have investigated the most recent set of post-2006 NHS reforms. In a study on management practice, Bloom *et al*. (2010) find a correlation between competition and hospital management quality, and a correlation between higher management quality and lower AMI mortality. To address concerns about endogeneity between market structure and management performance in their cross-sectional analysis, they use a measure of local political vulnerability as an instrument for the number of hospitals, based on the idea that politically vulnerable jurisdictions tend not to impose unpopular hospital closures. Several studies look more directly at patient outcomes during the post-2006 period of reform, including an earlier version of our work on AMI mortality (Cooper *et al*., 2010*b*2010b), and a study of the effects of competition on patients’ length of stay in hospital (Cooper *et al*., 2010*a*2010a). Gaynor *et al*. (2010) also look at the effect of competition on AMI mortality and trust-level overall mortality. These latter articles use DiD related methodologies and find that higher competition is associated with better hospital performance.

## 2. Patient Choice and Hospital Competition Reforms and Our Estimation Strategy

### 2.1. Competition in the English NHS

The English NHS is a publicly funded health system that is free at the point of use. In the 20 years prior to the reforms, patients had little choice over where they received care. From 1997 to 2002, the buyers of care (local government organisations) and providers of care (NHS-owned facilities) were tasked with working ‘cooperatively’ to organise care for their local communities (Klein, 2006). In practice, this involved coordinating care packages and negotiating annual contracts that were based on quality, volume and price.

The recent wave of NHS reforms were introduced in several stages from 2002 to 2008 and focused on increasing patient choice and hospital competition in order to create financial incentives for providers to improve their quality and efficiency (Department of Health, 2004,2009). The reforms involved changes to the demand side and supply side, as well as additional reforms to fundamentally restructure how hospitals in England were funded. Broadly, the reforms were designed to give patients choice over where they went for care, together with a reimbursement system where money followed the users’ choices, so that hospitals only received funding if they were able to attract patients. Hospitals were given increased managerial and fiscal autonomy, encouraged to compete on non-price elements of service and care and paid a fixed price based on a national tariff for different diagnoses that were drawn up by the Department of Health. In effect, the reforms created an incentive for hospitals to attract patients and compete with each other for volume in a market that only allowed providers to differentiate themselves on quality rather than price.

**Figure 1:**
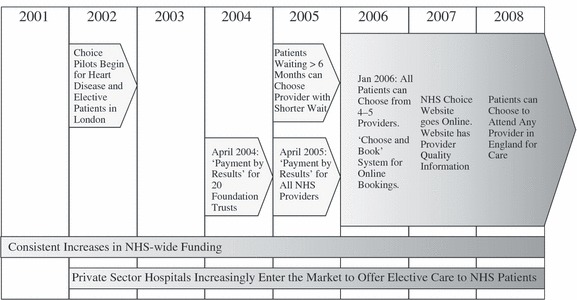
Timeline for the Second Wave of NHS Market-based Reforms (2001–8)

Figure 1 is a timeline of the key elements of the reforms. The market-based reforms occurred during a period when there was a significant surge in NHS spending and succeeded a wave of heavy performance management that focused on shortening waiting times. However, these funding changes and the performance management programme likely had a general effect across England, whereas the impact of the market-based reforms, which we study, will be much more market dependent.

Prior to 2002, patients themselves could not select where they went for secondary care and were largely confined to providers within their own Primary Care Trust. From 2002 to 2006, a small subset of patients who were waiting for long periods of time were allowed to opt to travel further for care but the majority of patients were largely confined to attending their local hospital and providers had no financial incentive to expand their market share. On 1 January 2006, all patients in England were formally given the ability to choose where they received elective care (Department of Health, 2009b; Dixon *et al*., 2010). However, it did take some time for the policy to bed in and for NHS Choose and Book, the electronic referral system, to become fully active (Dixon *et al*., 2010). Therefore, we take mid-2006 after the beginning of the new financial year as the key point when hospitals in England were significantly exposed to the financial incentives created by competition.

To create an environment that would support competition, beginning in 2002, the health service began paying for NHS patients to receive care in private sector facilities and attempted to diversify the hospital sector (Department of Health, 2002). The NHS helped to coordinate the development of Independent Sector Treatment Centres (ISTCs), which were to compete against traditional NHS hospitals to provide elective surgery and diagnostic services). Furthermore, in an effort to encourage local innovation, the government gave high performing hospitals additional fiscal, clinical and managerial autonomy. Hospitals that earned additional autonomy were referred to as ‘Foundation Trusts’ (FTs) (Department of Health, 2005).

In 2004 and 2005, the government implemented a new fixed-price funding mechanism called ‘Payment By Results’ (PBR), which was a case-based payment system modelled on the diagnosis-related group (DRG) system from America (Department of Health, 2009*a*2009a). Under PBR, hospitals were rewarded for increasing their activity and attracting more patients. The new reimbursement system allowed money to follow patients’ choices so providers were paid for their elective care based on the number of patients they were able to attract. Ultimately, since GPs are highly active in informing the destination of most referrals, GPs now play a substantial role dictating how money flows around the post-2005 NHS.

Along with giving patients a formal choice of where they receive secondary care in 2006, the government also introduced a paperless referral system (Department of Health, 2009*a*2009a). The booking interface included the ability to search for hospitals based on geographic distance and see estimates of each hospital’s waiting times that were based on the last 20 appointments at each facility. The ‘Choose and Book’ system was rolled out as patients in the NHS were given a choice of their secondary care provider. In addition, the government also created a website to provide additional quality information to inform patients’ choices. The hope was that providing information to patients would help them to make informed choices based on quality. The website currently includes detailed information on various aspects of provider performance, including risk-adjusted mortality rates, hospital activity levels, waiting times and infection rates sorted by procedures (Department of Health, 2009*c*2009c).

### 2.2. Hypothesis

From mid-2006 onwards, faced with fixed price reimbursements, increased elasticity of demand and the start of a new financial year, we expect that hospitals located in more competitive markets to take steps to differentiate themselves from one another on non-price aspects of their care, and in particular, by improving their clinical performance. The existing literature from the US suggests that fixed price hospital competition can prompt hospitals to improve their performance (Kessler and McClellan, 2000; Kessler and Geppert, 2005). We expect a similar response from English providers.

There are reasons to expect that NHS providers should be particularly responsive to this type of quality competition. First, NHS hospital managers face significant pressure to maintain annual financial surpluses, which would quickly be eroded if they failed to attract sufficient market share in the market for elective care and lost ground to other competing providers. Indeed, the NHS has embedded explicit rewards for high performing providers that maintain surpluses and rewards them with greater financial and managerial autonomy in the form of granting them FT status. In contrast, poorly performing hospitals have, in the past, actually had their senior management removed by the central government. Second, the incentives during the second period may be particularly sharp because GPs, who serve as patients’ agents, can now more easily refer patients to a wider range of hospitals. Elsewhere, Klein and Leffler (1981), Shapiro (1983) and Allen (1984), have found that even in markets with imperfect information, there is likely to be an equilibrium with optimal quality if consumers can perceive quality *ex post* and providers have an interest in attracting repeat business. Since GPs serve as agents for different patients for the same set of conditions on an ongoing basis, they are well positioned to observe quality *ex post* and use that information to advise future patients. In effect, despite the fact that patients seldom attend hospitals for the same procedures twice, GPs will be able to take advantage of their knowledge of their previous patients’ experiences and outcomes to inform future referrals.

Therefore, we expect that AMI mortality will decrease more quickly in more competitive markets from mid-2006 onwards after hospitals were exposed to competition created from the new NHS reimbursement system and the expansion of patient choice. While providers are not explicitly competing for AMI patients because competition in the NHS is limited to the market for elective care, we expect the market-based reforms to result in across-the-board improvements in hospital performance, which in turn will result in lower AMI death rates. To that end, Bloom *et al*. (2010) looked at NHS hospitals and found that better managed hospitals had significantly lower AMI mortality and that greater hospital competition was associated with better hospital management. Indeed, they observed that a one standard deviation improvement in a hospital’s overall management quality was associated with 0.66-percentage point reduction in AMI mortality and that better managed providers were able to attain more revenue per hospital bed and had higher patient satisfaction. Consistent with their findings, we expect that competition will prompt providers to improve their overall hospital management, which will result in across-the-board improvements in clinical performance. We capture these across-the-board improvements using risk-adjusted 30-day AMI mortality, our measure of hospital quality (which we discuss in more detail below).

### 2.3. Specification of Our Empirical Model

Our analysis centres on using regressions based on changes in AMI mortality trends to test whether hospitals located in more competitive markets improved their performance post mid-2006, relative to hospitals located in less competitive markets. Whereas, the bulk of the research on hospital competition relies on analysing a cross-sectional relationship between measured competition and quality, we use our estimates of market structure to determine which hospital markets were ‘treated’ and therefore exposed to the full force of the NHS market-based reforms after they were introduced.

Our research design is therefore DiD in style. However, the NHS market-based reforms that we are investigating do not fit neatly within the traditional DiD framework. In particular, every area in England was exposed, to some degree, to the reforms so, in principle, there are no distinct treatment and control groups. In practice, however, the NHS choice reforms will have had varying impact intensity across the country depending on the underlying geographical relationships between hospitals and residential areas. We assume that hospitals located in areas where choice is not geographically feasible will be subjected to less sharp financial incentives created from competition in comparison to hospitals located in areas where patients have considerable potential choice. Our DiD identification strategy is therefore based on the premise that the incentives from hospital competition are more intense in the period after the introduction of the NHS choice reforms and increasingly so for hospitals located in less concentrated markets. Similar DiD estimation strategies have been used to evaluate the employment effects of minimum wage increases (Card, 1992) and to study the 1990s internal market NHS reforms (Propper *et al*., 2008).

The second modification to the standard DiD setup is that, rather than comparing the levels of quality in the pre and post-policy periods, we estimate the effects of the reforms from a break in the time trend in AMI mortality after the mid-2006 ‘policy-on’ date. In our preferred empirical specification, we implement this using two-part, quarterly splines, split at the end of the second quarter in 2006 and interacted with our measure of potential market structure. We adopt this approach to illustrate explicitly that there are no differences in trends between high and low competition markets prior to the reforms. Note, there is no theoretical reason to expect a discrete jump in hospital quality in the first quarter after the policy-on date in the context of the NHS reforms and our spline specification imposes this restriction whilst allowing for a more gradual improvement in quality as the reforms begin to bite. However, as described below, we test and relax this restriction in our robustness tests.

Our general empirical regression specification is therefore:

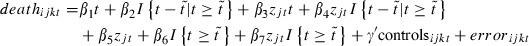
1

Here, *death*_*ijkt*_ is an indicator for whether patient *i*, from GP market *j*, treated at hospital site *k* died within 30 days of admission for AMI in period *t*. Subscript *t* indicates a running counter of quarters since quarter 1, 2002 (the first period in our data), and 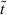 is the break point in the spline, corresponding to our policy-on period starting the end of the second quarter of 2006. Variable *z*_*jt*_ is the market structure index of GP market *j* at time *t*, which we describe in Section 3.3 below.^5^

We can estimate various alternative specifications by imposing restrictions on these parameters. Setting *β*_1_ = *β*_2_ = *β*_3_ = *β*_4_ = 0 gives rise to a standard DiD specification with continuous treatment variable, in which coefficient *β*_7_ is the estimated effect of the policy on the change in death rates between the pre and post-policy periods. Imposing the restrictions *β*_7_ = *β*_6_ = 0 instead gives our preferred spline-based difference-in-trends estimator in which *β*_4_ is the effect of the policy on the annual rate of change in death rates. Relaxing all these restrictions gives a combination of these two estimators, allowing for a step change at the policy-on date and a change in trends.

Our preferred specification is the one that imposes *β*_7_ = *β*_6_ = 0, but we report on the others in our robustness checks. In this preferred specification, coefficient *β*_1_ captures the baseline rate of decline in AMI mortality prior to the 2006 reforms, for locations in which our index of market structure is zero (a monopoly). Coefficients *β*_1_ + *β*_2_ capture the baseline rate of mortality decline in these low-competition places after reform.

Now consider a comparator place where there is high competition. The sum of *β*_1_ + *β*_3_ equals the time trend in mortality in these areas before the reform. The sum *β*_1_ + *β*_2_ + *β*_3_ + *β*_4_ is the time trend in mortality in highly competitive areas after the 2006 reform. The second partial derivate of the death rate trend with respect to differences in competition in the post-policy period is *β*_4_. This is our coefficient of interest and is a DiD estimate of the effect of competition on the trends in mortality.^6^ The coefficient *β*_3_ is also informative, in that it provides the basis to test for the existence of pre-policy differences in trends between high and low competition places *β*_3_ ≠ 0. The existence of pre-policy differences in trends would undermine the credibility of the DiD strategy.

We estimate (1) using Ordinary Least Squares and cluster our standard errors at the GP level to allow for error correlation across patients within GP markets. Note that the specification in equation (1) includes a vector of control variables as discussed in the data section and can be generalised to include hospital and GP fixed effects. Our specifications further include an interaction between Strategic Health Authorities (SHAs) and time trends, controlling for trends associated with local SHA policies and changes in regional funding.^7^

## 3. Data, Our Measures of Competition, Our Quality Indicator and the Instrumental Variable Strategy

### 3.1. Data Sources and Setup

Our article relies on patient-level Hospital Episodes Statistics (HES) data from 2002 to 2008. In addition to observations for patients with an emergency AMI, our analysis contains data on patients undergoing elective hip replacement, knee replacement, knee arthroscopy, cataract repair and hernia repair, which we use in the construction of our competition indices. At the hospital level, we know hospital site postcodes, the NHS trust to which the site belongs, and we have indicators of the hospital type (teaching hospitals, FTs status) and hospital size. Our work improves on previous research by using the hospital site-specific locations, rather than the trust headquarters. There are typically multiple treatment sites for each trust, separated by distances of up to 50 km, so using Trust locations provides only a very approximate indicator of the location at which treatment is carried out.^8^

We use GP and hospital site postcodes to calculate distances between patients’ GPs and the hospital where care was delivered. This distance is a key component in our analysis and is used as an input into most of our competition measures. For our main analysis, we use matrices of straight-line distances between GPs and NHS sites. For some of our supplementary results, we calculate origin-destination matrices from minimum road travel times along the primary road network.^9^

### 3.2. Measures of Health Care Quality

Our measure of hospital quality is the 30-day mortality rate for patients with an AMI.^10^ In our analysis, we include every patient who had a main International Classification of Disease 10 code of I21 or I22 and only include emergency AMI admissions and admissions where the patients’ length of stay was three days or more (unless the patient died within the first three days of being admitted) (World Health Organization, 2009).^11^

We chose to use AMI mortality as our measure of performance for four primary reasons. First, AMIs are a relatively frequent, easily observable medical occurrences that are clinically identifiable and have a substantial mortality rate. For example, in 2008 the overall, 30-day mortality rate for emergency AMI was 11.7% compared to a mortality rate of 0.20% for elective hip replacement. Second, with AMIs, there is a clear link between timely and high-quality medical intervention and patients’ survival (Bradley *et al*., 2006; Jha *et al*., 2007). Contrast this with a quality indicator such as readmissions for elective hip replacements, where a patient failing to stick to a rehabilitation programme after they were discharged could produce poor outcomes or lead to a readmission. Third, unlike other measures of performance, like hospital waiting times, AMI mortality (and death rates in general) are not subject to gaming or manipulation by hospitals. Fourth, AMIs are an emergency procedure where patients are generally taken directly to their nearest provider for care with little discretion over which hospital they attend, which mitigates hospitals ability to risk-select healthier patients for care. The fact that AMI is a non-elective procedure also mitigates biases due to the endogeneity of market structure to elective quality. This point is illustrated in Appendix A.

A further impetus for using AMI mortality is that it is frequently used by governments and private organisations to rank and compare hospital performance (including by the UK government).^12^^,^^13^ Consequently, 30-day AMI mortality is also often used in the academic literature as a measure of overall hospital performance in the UK and the US (Kessler and McClellan, 2000; Volpp *et al*., 2003; Propper *et al*., 2004,2008; Kessler and Geppert, 2005; Bloom *et al*., 2010; Gaynor *et al*., 2010; Propper and van Reenen, 2010). Consistent with its use as a measure of hospital performance, a recent study assessing the relationship between hospitals’ management quality and their overall performance found a statistically significant relationship between overall hospital management performance and hospital level 30-day AMI mortality (Bloom *et al*., 2010). Likewise, according to data made publicly available by Dr. Foster Health, despite accounting for less than 3% of total hospital deaths, standardised AMI mortality in English hospitals was positively correlated (*r* = 0.33) with overall hospital mortality for the financial year beginning in 2009.^14^ Likewise, in our administrative data, we have found that raw AMI mortality is positively correlated with elective hip and knee replacement waiting times (*r* = 0.33) and positively correlated with length of stay for elective hip and knee replacement (*r* = 0.11 and *r* = 0.22, respectively).

While 30-day AMI mortality is a frequently used measure of hospital quality, there are several issues with its use. First, as with all quality measures, despite being correlated with other dimensions of performance, there is a question of whether or not a single measure can capture the multi-dimensional nature of health care quality (McClellan and Staiger, 1999). A second issue with 30-day mortality is the noise inherent with this type of measure. This noise is particularly acute when researchers use hospital level data, where it is difficult to suitably risk adjust and hospital performance can vary from year to year. Our use of patient-level data, which allows for controls for patients’ socioeconomic status, age and co-morbidities, mitigates this problem. In our estimation, we control for co-morbidities using the Charlson co-morbidity index (Charlson *et al*., 1978) and control for patients’ socio-economic status using the income vector of the 2007 Index of Multiple Deprivation, which we include at the Census Output Area level (Communities and Local Government Department, 2009).^15^^,^^16^

### 3.3. Market Measures and Estimates of Market Structure

Identifying the impact of competition in the wake of NHS reforms requires accurately measuring market structure. In this article, we estimate market structure in the English NHS using both counts of providers and Herfindahl-Hirschman Indexes (HHIs) calculated using actual and predicted patient flows. Our aim in developing a range of measures of market structure is to illustrate that our results are robust across a number of measures of market structure, since there is not a single, agreed upon measure that is immune to each and every form bias.

The debate over measuring market structure centres around thwarting potential endogeneity between hospital quality and market structure, avoiding measures of market structure that simply reflect urban population density and defining a market size that accurately reflects the choice sets available to NHS users. Concerns over the endogeneity between measures of market structure and firm performance have been frequently cited in the literature and stem from three aspects of the construction of the market structure measures.

These different forms of bias could positively or negatively affect our estimates. First, the physical market size itself could be associated with hospital performance, which would bias our estimates upwards. For example, a high-quality provider might attract patients from a larger area, and hence appear to be operating in a less concentrated market. Second, the actual patient flows that are used to estimate market shares (and form the key component of the HHI) could be associated with quality because high quality providers could attract all the local business and as a result appear to be operating in more concentrated markets and bias our estimates downwards. Third, the actual location of hospitals and of new market entrants may be associated with performance. For example, if new hospitals were reluctant to locate near high quality providers, this would artificially show high quality providers to be operating within concentrated markets and bias our estimates of the treatment effect downwards.

In addition to concerns about endogeneity, there are also fears that the various measures of market structure will be spuriously correlated with urban population density, which stem from two causes. First, densely populated cities have more hospitals within smaller geographic areas, and as a result, urban areas will likely appear more competitive. Second, measures of market structure that are calculated within fixed geographic markets may be biased because the time it takes to travel 30 km in an urban area will differ significantly from the time it takes to travel 30 km in a rural area.

In our estimates of market structure, we calculate competition within the market for elective secondary care for NHS funded patients. We focused on competition for elective care because this was the only hospital market where competition occurred during the time period we are studying. We study five high volume procedures – hip replacement, knee replacement, arthroscopy, hernia repair and cataract repair – and develop composite measures of market structure, which are weighted averages of the competition measures that we calculated for each of the individual procedures. The bulk of our measures of market structure are based on actual patient flows. However, Kessler and McClellan (2000) have suggested that any measures of market structure based on actual patient flows could be endogenous to hospital quality because they may be correlated with various unobserved characteristics of either patients or providers. As a result, in addition to using an instrumental variable strategy, we also estimate a measure of market structure, similar to the measure used in Kessler and McClellan (2000), which is based on predicted patient flows generated from models of patient choice.

We centre all of our markets on GP practices, rather than on hospitals, because this mirrors the post-2005 NHS market structure, where patients select their hospital in conjunction with their GP (Dixon *et al*., 2010). In addition, were we to centre our measures of market structure on hospitals, then there is the risk that if unobserved determinants of hospital choice are correlated with patient characteristics, there could be spurious and problematic associations between health status and market structure.

To measure market concentration using actual patient flows, we calculate the negative natural logarithm of an HHI (*nlhhi)* based on hospitals’ market shares. This transformation is convenient because the *nlhhi* increases with competition, with zero corresponding to monopoly and infinity to perfect competition. In addition, this measure is equivalent to the natural log of the number of equal size firms in the market, which makes interpreting the index more intuitive. Thus, for given market area *j,* our concentration index is:

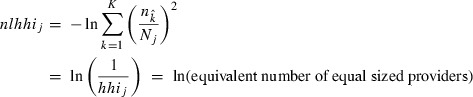
2

Here, *n*_*k*_ is the number of procedures carried out at hospital *k* within market *j* and *N*_*j*_ is the total number of procedures carried out in market *j*. Note that *n*_*k*_ includes procedures performed at hospital *k* that were not referred from market *j*.

We construct our preferred market definition as follows: consider an elective procedure, e.g. hip replacements, in one year, e.g. 2002. We use matrices of patient flows from GP practices to hospitals for hip replacement in 2002 to deduce GP-centred markets. Specifically, we find the radius that represents the 95th percentile of distance travelled from a GP practice to hospitals for hip replacements in 2002. This defines the *feasible* choice set for patients at this GP practice in 2002. We then compute the HHI based on *all* hospitals providing hip replacements within this GP’s market, regardless of whether this GP actually refers patients to all of these hospitals. This process is repeated for all GPs, for all years 2002–8 and for all five key elective procedures. A single elective HHI is calculated for each GP per year as a weighted average of the procedure-specific HHIs with weights proportional to the volume of patients in each procedure category.^17^

In addition to calculating this HHI within a variable radius market, we also compute a number of alternative HHIs using other market definitions. These include an HHI measured within a fixed radius market, which is derived in a similar way to the variable radius HHI described above, except that we use a fixed 30 km radius drawn around each GP practice in the country to delineate the market boundaries. The second alternative index is an HHI based on travel times along the primary road network from each GP. Here, we include hospitals in our relevant markets if they fall within a 30-min car ride from a referring GP. A third alternative is based on our 95% variable radius market but it does not treat sites within the same trust as competitors and only views sites from a different Trust as viable alternatives in the calculation of our HHI. We have also calculated our preferred purchaser-perspective measures using the count of hospitals within each market in lieu of using HHIs. In addition, we calculate one measure of market concentration from the provider’s perspective, where the market is centred on hospitals and the market is defined a fixed radius of 20 km drawn around each site.

Alongside the HHIs we generated using actual patient flows, we also created an HHI derived from predicted patient flows that is based on the strategy used in Kessler and McClellan (2000). Building our predicted patient flow HHI is a two-step procedure. The first step involves estimating a patient choice model based on hospital and GP locations, and hospital and patient characteristics.^18^ From this step we predict the number of patients each GP refers to their local hospitals, controlling for patient and provider characteristics and GP-hospital differential distances. We then use these numbers to generate the HHIs.^19^ However, whereas Kessler and McClellan (2000) used a conditional logit to model patient choices, we use a Poisson regression on aggregate GP-hospital flows, which is equivalent but is simpler to compute (Guimaraes *et al*., 2003).

As Table 1 illustrates, all these measures of GP-centred competition are moderately correlated. The indices from fixed radius and time-based market definitions are highly correlated. Indices based on market definitions using GP hospital flows are quite highly correlated with each other and only moderately correlated with the fixed distance and time-based indices. We favour the variable radius methods that infer markets from de-facto patient choices over hospitals, not least because this is less correlated with urban density.^20^

**Table 1:** *Correlations Between Different Measures of Market Structure*

	−log(HHI)−95%	−log(HHI)−30 km	−log(HHI)−30 min	−log(HHI)-predicted flows	Mean	Standard deviation
−log(HHI)-95%	1.00				0.7483	0.5639
−log(HHI)-30 km	0.48	1.00			1.4860	0.9053
−log(HHI)-30 min	0.43	0.92	1.00		1.2686	0.8081
−log(HHI)-predicted flows	0.47	0.92	0.86	1.00	1.0458	0.5930

*Notes*. HHI, Herfindahl-Hirschman Indexes.

As a further check of robustness, we estimate (1) substituting an indicator variable for our competition measure, which is equal to one if a patient’s GP practice is located in an urban area.^21^ For further robustness, we also reconstruct the competition index using the shares of secondary school pupils in schools within our GP-centred markets (defined by the 95% referral radius during the pre-policy period) for use in a placebo test. These tests are designed to confirm that our results are driven by competition, rather than spurious associations with urban density.

### 3.4. Instrumental Variable Estimation

In an effort to thwart the endogeneity that we described earlier, in addition to creating HHIs from predicted patient flows, we have also developed an instrument for competition. Our preferred instrument takes advantage of the historically determined hospital locations in England and is based on the variation in distance to a patient’s nearest four hospitals. Specifically, our instrument for market structure is the standard deviation of distances from GPs to their nearest four hospitals, conditional the on the distance to the patient’s nearest hospital (a control which we introduce in order to control for potential urban/rural differences in GP location). This IV strategy rests on the fact that NHS hospital and GP *relative* positions are unrelated to hospital quality, which is supported by the fact that hospital locations in England are largely a historical artefact which have not changed substantially since the NHS was founded in 1948 (Klein, 2006).

To illustrate our IV strategy, imagine two hospital markets centred on two individual GP practices (*A* and *B*). The nearest provider in the area of *GP*_*A*_ is located at 5 km, and the remaining three at 15, 20 and 30 km. The nearest provider to *GP*_*B*_ is also at 5 km, but with the remaining three all within 10 km (in different directions). In this situation, while the distance to the nearest provider is the same in both cases, the alternatives available to patients of *GP*_*B*_ are much more substitutable than the alternatives available to patients of *GP*_*A*_ because they are all within a similar travel distance, so patients of *GP*_*A*_ are much more likely to attend the nearest provider. We therefore assume that GP-centred markets characterised by a high dispersion in distances to local providers are low choice and therefore low competition markets.

In practice, we have three instrumented variables, which include the baseline measure of market structure, the pre-policy time trend interacted with market structure and the post-policy time trend interacted with market structure. We perform our IV with a 2SLS estimator and include GP and hospital fixed effects.

## 4. Results

### 4.1. Empirical Results

Our estimation sample contains 433,325 patients who had an AMI between 2002 and 2008. There are 227 hospital sites providing care for AMI for patients who were registered at 7,742 GP practices. Hospital quality, measured by 30-day AMI in-hospital mortality, improved consistently from 2002 to 2008, as shown in Table 2. Likewise, the number of AMIs treated per year also fell. This reduction in mortality and reduction in overall AMI occurrences is consistent with international trends and is driven, in part, by increasing adoption of new technology in the treatment of AMI and improvements in public health (Committee on Second Hand Smoke Exposure and Acute Coronary Events, 2009; Meyers *et al*., 2009; Schroeder, 2009; Walker *et al*., 2009). The coefficient of variation in 30-day mortality rates between hospitals was approximately 30% per year, suggesting that there is significant variation in outcomes between providers.

**Table 2:** *Thirty-day Patient-level AMI Mortality from* 2002 *to* 2008

Year	Population treated	Mean mortality	Standard deviation
2002	64933	0.1563	0.3631
2003	64776	0.1506	0.3576
2004	66226	0.1415	0.3485
2005	62433	0.1396	0.3465
2006	59760	0.1309	0.3373
2007	59017	0.1247	0.3304
2008	56180	0.1196	0.3245
2002–8	433,325	0.1381	0.3451

Observations are limited to patients between 39 and 100 years of age with a length of stay greater than two days, treated at sites that treated more than 99 AMIs per year. Unlike the regressions that we present, we do not limit the distance that patients travelled for care. AMI, acute myocardial infarction.

Table 3 provides OLS estimates of the DiD specification of (1) using our preferred empirical specification and index of market structure (the *nlhhi* using the 95% GP market described in Section 3.3 and restricting β_6_ = β_7_ = 0 as discussed in Section 2.3). The variables of interest in our sample are described in Appendix B. Our main interest is in the coefficient on the interaction between the 2006–8 trend and our market structure index. This coefficient is *β*_4_ in (1) and it estimates the impact of our policy by measuring the effect of greater competition on the quarterly reduction in AMI mortality after patient choice and competition were introduced in 2006.

**Table 3:** *Least Squared Estimates* of (1)*with Market Structure Measured as the nlhhi Within a Market That Captures all Hospitals Within the 95th Percentile of each GP’s Maximum Travel Distance*

	(1)	(2)	(3)	(4)	(5)
*2002–5 Trend*	−0.0018^***^	−0.0026	−0.0024^***^	−0.0023^***^	−0.0024^***^
	(0.0002)	(0.0002)	(0.0002)	(0.0002)	(0.0002)
*2006–8 Trend*	−0.0004^**^	−0.0014^**^	−0.0014^**^	−0.0014^**^	−0.0014^**^
	(0.0004)	(0.0004)	(0.0004)	(0.0004)	(0.0004)
*2002–5 Trend × nlhhi*	0.0004^*^	0.0003	0.0002	0.0001	0.0002
	(0.0002)	(0.0002)	(0.0002)	(0.0002)	(0.0002)
*2006–8 Trend × nlhhi*	−0.0013^**^	−0.0013^**^	−0.0014^**^	−0.0013^**^	−0.0014^***^
	(0.0004)	(0.0004)	(0.0005)	(0.0004)	(0.0005)
*nlhhi*	−0.0017	0.0020	−0.0015	−0.0014	−0.0015
	(0.0023)	(0.0022)	(0.0028)	(0.0027)	(0.0028)
Patient characteristics	No	Yes	Yes	Yes	Yes
Hospital fixed effects	No	No	Yes	No	Yes
GP fixed effects	No	No	No	Yes	Yes
*N*	422,350	422,350	422,350	422,350	422,350
R^2^	0.036	0.105	0.126	0.125	0.126

* Significant at 5% level.

** Significant at 1%.

*** Significant at 0.1%. Dependent variable = 1 if patient died within 30 days of their admission to hospital following an emergency AMI. Hospital characteristics: hospital type (foundation trust, teaching hospital or traditional acute hospital), number of AMIs treated at the hospital per year. Patient characteristics: age, gender, Charlson comorbidity score and patient socioeconomic status measured using the income component of the 2004 Index of Multiple Deprivations at the output area. Standard errors are clustered on GP-practices. AMI, acute myocardial infarction.

Table 3 reports several versions of our preferred specification, where we have included and excluded patient characteristics, hospital and GP fixed effects. The results presented in Table 3 illustrate that our main finding is not highly sensitive to the control variables we include in our estimator. In each specification in Table 3, we find that after the formal introduction of choice in 2006, mortality decreased more quickly in more competitive markets. The coefficient of our interaction term is nearly identical in all specifications and it remains negative and significant with and without GP and hospital fixed effects or the exclusion of patient characteristics. Column (5) is our overall preferred specification and includes both GP and hospital fixed effects, which control for the possibility of changing GP, patient and hospital composition in high competition and low competition areas. Based on the coefficient of interest in Column (5) from Table 3, taking a one standard deviation gap in *nlhhi* (=0.565) as our benchmark, 30-day AMI mortality fell 0.31 percentage points faster per year after the reforms for patients treated in more competitive markets (=0.564 × 0.0014 × 4, because the time trends are quarters). Framed differently, the shift from a market with two equally sized providers to one with four equally sized providers after the reforms would have resulted in a 0.39 percentage point faster reduction in AMI mortality per year from 2006 onwards.

An essential observation from Table 3 is that the pre-policy trend in AMI mortality in areas with uncompetitive market structures is not statistically different from the trend in markets with competitive structures once we control for patient characteristics. The coefficient on the *2002–5 Trend × nlhhi* interaction is near zero and statistically insignificant in all specifications other than Column (1), which includes no control variables. This shows that these different markets were balanced in terms of the mortality trends pre-reform, and allays fears that the DiD results simply pick up pre-existing differences in trends. The full set of results from our overall preferred specification are presented in Appendix C.

Table 4 shows that the results we observed in Table 3 are not highly sensitive to the choice of which market structure index we use to define the treated groups.^22^ It presents OLS estimates of (1) using seven separate measures of market structure. Our findings remain consistent and significant across the seven different measures of market structure. The coefficient on the interaction between *market structure* and the 2006–8 trend is always negative and significant, illustrating that higher competition was associated with higher quality (lower mortality), regardless of how we quantified market structure. Column 3 includes estimates of competition where we fix the *nlhhi* in time as the average of the 2002–5 *nlhhis,* which uses pre-reform patient flows from a time period where patients had no choice over their provider (hence the patient flows are likely unrelated to quality). In addition, in Column (6) we have also presented a measure of market structure centred on hospitals. Finally, Column (7) includes estimates of our treatment effect where market concentration is measured using predicted patient flows similar to those used by Kessler and McClellan (2000).

**Table 4:** *Least Squared Estimates of* (1) *Using Seven Alternative Measures of Market Concentration*

	(1)	(2)	(3)	(4)	(5)	(6)	(7)
*2002–5 Trend*	−0.0024^***^	−0.0024^***^	−0.0022^***^	−0.0026^***^	−0.0026^***^	−0.0023^***^	−0.0028^***^
	(0.0002)	(0.0002)	(0.0002)	(0.0002)	(0.0002)	(0.0002)	(0.0002)
*2006–8 Trend*	−0.0018^***^	−0.0016^***^	−0.0020^***^	−0.0012^**^	−0.0015^**^	0.0005	−0.0014^**^
	(0.0004)	(0.0004)	(0.0004)	(0.0005)	(0.0005)	(0.0004)	(0.0005)
*2002–5 Trend × nlhhi*	0.0002	0.0003	0.0000	0.0003^*^	0.0003^*^	0.0002	0.0006^**^
	(0.0002)	(0.0002)	(0.0003)	(0.0001)	(0.0001)	(0.0002)	(0.0002)
*2006–8 Trend × nlhhi*	−0.0012^*^	−0.0015^**^	−0.0017^*^	−0.0009^**^	−0.0009^**^	−0.0009^**^	−0.0012^**^
	(0.0005)	(0.0005)	(0.0007)	(0.0003)	(0.0003)	(0.0003)	(0.0004)
*nlhhi*	–	−0.0026	−0.0022	0.0036	0.0013	0.0099	0.0091
	(0.0032)	(0.0042)	(0.0070)	(0.0067)	(0.0061)	(0.0073)
Patient characteristics	Yes	Yes	Yes	Yes	Yes	Yes	Yes
Hospital fixed effects	Yes	Yes	Yes	Yes	Yes	Yes	Yes
GP fixed effects	Yes	Yes	Yes	Yes	Yes	Yes	Yes
*N*	422,350	422,350	382,026	439,365	437,185	421,094	461,508
R^2^	0.126	0.126	0.127	0.126	0.126	0.126	0.124

* Significant at 5% level.

** Significant at 1%.

*** Significant at 0.1%. Column (1) *nlhhi *= negative ln of HHI within 95% variable market with competition measured as the average HHI between 2002 and 2005 prior to the reforms; column (2) *nlhhi *= negative ln of HHI within 95% variable market where competition is measured between hospital trusts, not sites; column (3) *nlhhi *= negative ln of HHI within 75% variable radius market; column (4) *nlhhi *= negative ln of HHI within fixed 30 km radius market; column (5) *nlhhi *= negative ln of HHI within market defined by 30-min drive time from each GP practice; column (6) *nlhhi* is centred on hospitals and defined within a 20 km fixed radius; column (7) *nlhhi =* negative log of HHI based on predicted patient flows. Dependent variable = 1 if patient died within 30-days of their admission to hospital following an emergency AMI. Hospital characteristics: hospital type (foundation trust, teaching hospital or traditional acute hospital), number of AMIs treated at the hospital per year. Patient characteristics: age, gender, Charlson comorbidity score and patient socioeconomic status measured using the income component of the 2004 Index of Multiple Deprivations at the output area.

Standard errors are clustered on GP-practices. HHI, Herfindahl-Hirschman Indexes; AMI, acute myocardial infarction.

In addition to using HHIs, in Table 5 we also present least squares estimates of (1) using logged hospital counts within each market definition to calculate market structure across the country. While counts are not as sensitive to the underlying market characteristics as an HHI, they do not rely on patient flows and serve as a robustness check on our HHI estimations. We calculate count measures of competition in four market definitions – two separate variable radius markets, a fixed radius market and a time-based radius market. The counts are logged so our estimates are more easily comparable to the *nlhhis*. Regardless of the count-based market structure measure that we use, we consistently find that the interaction term of interest is negative and significant, indicating that a competitive market structure was associated with a statistically significant reduction in AMI mortality after 2006 with estimates that are a similar magnitude to those we generated measuring market structure using HHIs.

**Table 5:** *Least Squared Estimates of* (1)*with Market Concentration Measured as the Natural Log of the Count of Hospitals Within Four Market Definitions*

	(1)	(2)	(3)	(4)
*2002–5 Trend*	−0.0025^***^	−0.0023^***^	−0.0026^***^	−0.0027^***^
	(0.0002)	(0.0002)	(0.0002)	(0.0002)
*2006–8 Trend*	−0.0013^**^	−0.0015^***^	−0.0012^*^	−0.0013^*^
	(0.0005)	(0.0004)	(0.0005)	(0.0005)
*2002–5 Trend* × *count*	0.0002	0.0001	0.0002^*^	0.0003^*^
	(0.0001)	(0.0002)	(0.0001)	(0.0001)
*2006–8 Trend* × *count*	−0.0009^**^	−0.0016^***^	−0.0008^**^	−0.0008^**^
	(0.0003)	(0.0004)	(0.0003)	(0.0003)
*count*	−0.0031	−0.0006	0.0049	0.0032
	(0.0019)	(0.0027)	(0.0058)	(0.0058)
Patient characteristics	Yes	Yes	Yes	Yes
Hospital fixed effects	Yes	Yes	Yes	Yes
GP fixed effects	Yes	Yes	Yes	Yes
*N*	422,350	382,026	439,365	433,699
R^2^	0.126	0.127	0.126	0.126

* Significant at 5% level.

** Significant at 1%.

*** Significant at 0.1%. Column (1) 95% variable market; column (2) 75% Variable market; column (3) Fixed 30 km radius market; column (4) market defined 30-min travel time from each GP. Dependent variable = 1 if patient died within 30-days of their admission to hospital after an emergency AMI. Hospital characteristics: Hospital type (foundation trust, teaching hospital or traditional acute hospital), number of AMIs treated at the hospital per year and patient characteristics: age, gender, Charlson comorbidity score and patient socioeconomic status measured using the income component of the 2004 Index of Multiple Deprivations at the output area. Standard errors are clustered on GP-practices. AMI, acute myocardial infarction.

To illustrate that our findings are the result of changes in hospital quality, rather than the by-product of different patient populations living in high *versus* low competition regions, we estimated (1) using *hospital × year* fixed effects. Hospital interactions on year fixed effects should capture improvements in quality from hospitals year to year. When we estimated (1) and included *hospital × year* fixed effects interactions, as anticipated, it washes out the effect of competition.

### 4.2. Test of Functional Form

Table 6 presents additional estimates of our treatment effect using different function functional forms discussed in Section 2.3, and shows some tests on the various parameter restrictions in the general model of (1). In addition to our preferred estimator, we estimate the treatment effect using a traditional DiD regression (Column 1), added the *post-policy* dummy and a *post-policy* * market structure interaction to our preferred spline estimator (Column 2), and shown the most general specification with year dummies and year dummies interacted with market structure (Column 3). Note the specification in Column 3 is set up so that the coefficients show the marginal change from the previous year.

**Table 6:** *Alternative Regression Specification with Market Structure Measured as the nlhhi Within a Market that Captures all Hospitals Within the 95th Percentile of each GP’s Maximum Travel Distance*

Standard difference-in-difference		Time trend interactions & post ^*^ *nlhhi* interaction		Year-post dummies ^*^ *nlhhi*	
*Post*	−0.0267^***^	*2002–5 Trend*	−0.0023^***^	*2003_Post*	−0.0072^*^
	(0.0020)		(0.0002)		(0.0032)
*Post* × *nlhhi*	−0.0038^*^	*2006–8 Trend*	−0.0013^**^	*2004_Post*	−0.0123^***^
	(0.0019)		(0.0005)		(0.0031)
*Nlhhi*	−0.0030	*2002–5 Trend* × *nlhhi*	0.0000	*2005_Post*	−0.0097^**^
	(0.0022)		(0.0002)		(0.0032)
		*2006–8 Trend* × *nlhhi*	−0.0015^**^	*2006_Post*	−0.0091^**^
(0.0004)	(00033)			
*nlhhi*	0.0004	*2007_Post*	−0.0027
	(0.0035)		(0.0035)
*Post*	−0.0024	*2008_Post*	−0.0082^*^
	(0.0029)		(0.0036)
*Post* × *nlhhi*	0.0029	*2003_Post* × *nlhhi*	−0.0023
	(0.0031)		(0.0036)
		*2004_Post* × *nlhhi*	0.0027
(0.0034)		
*2005_Post* × *nlhhi*	0.0025
	(0.0035)
*2006_Post* × *nlhhi*	−0.0015
	(0.0038)
*2007_Post* × *nlhhi*	−0.0077^*^
	(0.0038)
*2007_Post* × *nlhhi*	−0.0018
	(0.0037)
*nlhhi*	−0.0001
	(0.0031)
F-test p-value		All time trends	0.0000		
		Trends ^*^ *nlhhi*	0.0018		
		Post & post ^*^ *nlhhi*	0.4470		
Patient characteristics	Yes		Yes		Yes
Hospital fixed effects	Yes		Yes		Yes
GP fixed effects	Yes		Yes		Yes
*N*	422,350		422,350		422,350
R^2^	0.125		0.126		0.126

* Significant at 5% level.

** Significant at 1%.

*** Significant at 0.1%. Dependent variable = 1 if patient died within 30-days of their admission to hospital following an emergency AMI. Hospital characteristics: Hospital type (foundation trust, teaching hospital or traditional acute hospital), number of AMIs treated at the hospital per year. Patient characteristics: age, gender, Charlson comorbidity score, patient socioeconomic status measured using the income component of the 2004 Index of Multiple Deprivations at the output area. Standard errors are clustered on GP-practices. AMI, acute myocardial infarction.

As Table 6 illustrates, our estimates of the treatment effect of competition are not substantively dependent on the functional form of our estimator. The simple DiD estimator in column 1 shows a fall in mortality in high competition areas relative to low competition areas after the policy-on date. The estimates presented in the second column of Table 6 suggest that there was not a discrete jump in performance in 2006 and provides support for our preferred spline specification that is predicated on the presence of a more gradual improvement in performance. We provide some formal tests for these restrictions in Column 2. The joint test of significance of all the spline coefficients gives a p-value less than 0.01, the test for the two coefficients on the interaction between the splines and market structure index is 0.001. In contrast, the joint test of the coefficients on the *post-policy* dummy and *post-policy × market structure* interaction gives a p-value of 0.447. These tests provide evidence in favour of our spline-based, difference-in trends specification.

In the third column, with the most flexible non-parametric specification, the coefficients of interest on the *year × market structure* interactions are negative in 2006, 2007 and 2008. Although these last semi-parametric estimates are imprecise, the drop in the 2007 period is significant at 5% and the general pattern of point estimates is broadly in line with our main results.

**Figure 2:**
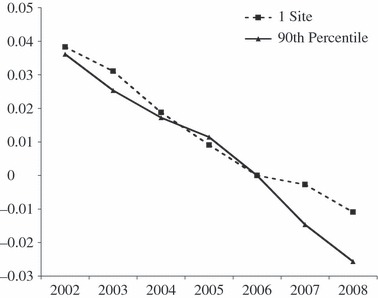
*Changes in Predicted Mortality Rates Over Time in Monopoly Markets with One-Site Providing Care and in Markets in the Most Competitive Decile of Our Market Structure Index*

Figure 2 plots the predicted mortality rates over time using the point estimates from this semi-parametric procedure, by way of illustrating the general pattern, which our estimates are trying to uncover. The dashed line shows the prediction for concentrated market structure locations (i.e. with the market structure index set to zero, corresponding to one hospital), which represent the counterfactual of what would have happened to AMI mortality in the absence of the reforms. The solid line shows the pattern for competitive market structures, with the index set to the top decile (*nlhhi =* 1.55). Both plots are normalised to zero in 2006. The plots show clearly that the trends tracked together prior to the reform date, but diverged from 2005/6 on. The picture supports our main finding that locations with less concentrated markets that were most exposed to the effects of the reforms sustained a higher and improved rate of decline in mortality rates than the counterfactual areas that remained less exposed to competition.

### 4.3. Other Tests of Robustness and Instrumental Variables Estimates

Table 7 presents robustness checks to illustrate that the effect we identify in our interactions between our post-2006 time trend and our measure of market structure are not simply spurious associations with urban density or driven by problematic (endogenous) associations between market structure and firm performance. In Column (1), we present interactions between the time trends and an indicator variable for whether or not the patient’s local hospital market is located in a city, substituted for the measure of market structure.^23^ The interaction term between the city indicator and our post-policy trend is not significant and is approximately half as large as our main estimate. In Column (2), we present a ‘placebo/falsification’ test in which we replace hospital market structure with a measure of market structure amongst state secondary schools. Clearly, if choice and competition in the health service drive our results, we would not expect to see a significant impact from schooling structure on AMI mortality rates in response to the NHS choice reforms. In contrast, if we are simply picking up changes in mortality trends in dense *versus* less dense places, then the market structure in schooling is just as likely to produce a ‘false positive’ result. Reassuringly, the coefficient on the interaction between post-reform trends and schooling structure is near zero and insignificant.

**Table 7:** *Additional Robustness Tests*

	Test of urban effect	School competition falsification test	Instrumental variable estimate
*2002–5 Trend*	−0.0025^***^	−0.0028^***^	−0.0030^***^
	(0.0004)	(0.0004)	(0.0005)
*2006–8 Trend*	−0.0004	0.0003	−0.0017
	(0.0007)	(0.0008)	(0.0010)
*2002–5 Trend* × *market structure*	0.00034	0.0002	0.0011
	(0.0004)	(0.0001)	(0.0006)
*2006–8 Trend* × *market structure*	−0.0007	−0.0002	−0.0031^*^
	(0.0008)	(0.0002)	(0.0014)
*Market structure*	–	–	0.0107
			(0.0261)
Patient characteristics	Yes	Yes	Yes
Hospital fixed effects	Yes	Yes	Yes
GP fixed effects	Yes	Yes	Yes
*N*	422,350	414,230	425,408
R^2^	0.126	0.126	0.105

* Significant at 5% level.

** Significant at 1%.

*** Significant at 0.1%. Dependent variable = 1 if patient died within 30-days of their admission to hospital following an emergency AMI. Hospital characteristics: Hospital type (foundation trust, teaching hospital or traditional acute hospital), number of AMIs treated at the hospital per year. Patient characteristics: age, gender, Charlson comorbidity score, patient socioeconomic status measured using the income component of the 2004 Index of Multiple Deprivations at the output area. Standard errors are clustered on GP-practices for the IV and falsification test. Standard errors are clustered on hospitals for the hospital centred fixed-radius HHI. AMI, acute myocardial infarction; HHI, Herfindahl-Hirschman Indexes.

The third column of Table 7 presents our instrumental variables estimates. We instrument market structure using variation of the straight-line distance from each GP to the nearest four elective providers, controlling for the distance to the patient’s nearest provider. The F-tests on our instrumented variables are significant (p < 0.001) with F-statistics of 207.32, 209.76 and 75.41, respectively for the *2002–5 × market structure* term, the *2006–2008 × market structure* term and the baseline *market structure*. In addition, the signs on the standard deviation coefficients in the first stage are negative suggesting that higher standard deviations are associated with lower *nlhhi*s. The IV estimates show a similar pattern to the OLS results in Table 3. The point estimate on our coefficient of interest is more than double that in the equivalent OLS specification, although the standard errors are also higher and the Hausman test indicates no statistically significant difference between the IV and OLS coefficient. There is no evidence from the IV estimates that it is the endogeneity of market structure to health service quality that drives our findings. Appendix D. includes the first stage estimates from our IV estimator.

## 5. Conclusions

There has been significant debate over the potential for hospital competition to improve hospital quality. This debate has been particularly intense in England, where two successive UK governments have experimented with introducing hospital competition into the tax-funded English NHS. Previous experience with hospital competition in England has not been positive. Looking at the 1990s internal market, Propper *et al*. (2004,2008) found that higher competition was associated with higher AMI mortality. This is consistent with speculation that in markets where hospitals can compete on price and quality, price is likely to decrease but so too is quality (Gaynor, 2004).

This article looks at the more recent wave of market-based reforms in the English NHS. In the latest wave of reforms, patients were given the ability to select their secondary care provider, prices were regulated by the UK Department of Health, and hospitals could only compete on quality. We exploit the introduction of patient choice in 2006 to determine whether increases in hospital competition in a market with fixed prices led to improvements in hospital quality. Consistent with previous work examining the relationship between competition and quality, we measure hospital quality using 30-day mortality from AMI.

In our analysis, we find that higher competition was associated with a faster decrease in 30-day AMI mortality after the formal introduction of patient choice in January 2006. Our results are robust to a number of specifications and definitions of market concentration and consistent with our tests of the counterfactuals. The title of our article asked whether or not hospital competition saved lives. Judging from the impact of the reforms on 30-day AMI mortality, the reforms did save lives. Based on the results from our preferred specification, we can provide an indicative estimate that the reforms resulted in approximately 300 fewer deaths per year after the reforms were introduced in 2006 (based on a mean *nlhhi* of 0.748, an average 70,000 AMI cases in each year, and the coefficient in Table 3 Column (4): 70,000 × 4 × 0.0014 × 0.748). Crucially, this estimates is for lives saved by reducing AMI mortality alone and ultimately, AMI mortality only accounts for approximately 0.5% of total NHS hospital admissions. So, given that we postulate that AMI mortality is correlated with quality across hospitals, in practice, the lives saved from the reforms when estimated across the NHS and all dimensions of service provision are likely to be significantly higher.

We posit that the improvements we observe in hospital quality were driven by increases in competition for elective care. Competition in the elective market in England likely prompted hospitals to take a number of steps to improve clinical performance, such as undertaking clinical audits, tightening clinical governance, making investments in new technology and improving hospital management. Those improvements spurred on by elective competition likely resulted in across-the-board improvements in hospital quality. These general quality improvements, we argue, are likely captured by our chosen indicator of quality, 30-day AMI mortality, where there is a close link between timely and effective medical interventions and patient survival (Bradley *et al*., 2006; Jha *et al*., 2007).

Thus, our results suggest that, in contrast to what Propper *et al*. (2008) observed for the 1990s internal market, competition in the current fixed price market did save lives. These results are consistent with Kessler and McClellan (2000) and Kessler and Geppert (2005) that were focused on hospital competition in the US and found that hospital competition within a market with fixed prices led to an increase in hospital quality, as indicated by a reduction in AMI mortality. Our results add support to current efforts in England to increase the amount of publicly available information on quality and promote hospital competition in the absence of price competition.

The conclusion, then, is that hospital competition, introduced in a fixed priced market, can lead to an increase in the quality of hospital services, as economic theory would predict. The rise in quality we have observed in the wake of the most recent NHS reforms has undoubtedly increased consumer welfare. We postulate that given the level of quality improvements that can be attributed to these reforms these results are consistent with an overall improvement in social welfare. However, more research needs to be carried out to evaluate this latter assertion empirically.

## Footnotes

1 According to the most recent OECD data recorded for 2008, health care spending as a percentage of GDP was 16.0% for the US, 10.4% for Canada, 11.2% for France, 10.5% for Germany, 9.9% for the Netherlands and 8.7% for the United Kingdom (OECD, 2010).

2 In the health care sector, these have included policies to increase the choice of health insurers in the Netherlands, policies to increase choice and introduce competition into Medicare prescription drug coverage for seniors in the US, and efforts to expand patients’ choice of health care providers in England, the Netherlands, Sweden and Denmark.

3 See Kessler and McClellan (2000); Propper *et al*. (2004,2008); Kessler and Geppert (2005); Gaynor *et al*. (2010).

4 Looking at competition in a market with unregulated prices, Gowrisankaran and Town (2003) found that higher competition led to lower AMI mortality, which served as a proxy for overall quality. Likewise, Sari (2002) uses hospital complication rates as a proxy for quality, and finds that higher hospital competition led to improvements in quality. Hamilton and Ho (2000) looked at competition in a variable priced market by examining hospital mergers and found that there was no significant relationship between competition and mortality. Volpp *et al*. (2003) examine price competition in New York and find that it is associated with significant increases in AMI mortality.

5 This index is calculated from data for the whole calendar year, rather than the quarter.

6 This is easily deduced since:

7 There are ten NHS Strategic Health Authorities (SHAs) in England; each represents a different region of the country. SHAs are responsible for implementing the policy that is set by the Department of Health and managing local health care provision. Increasingly, over the period we study, policy making has been devolved to local SHAs.

8 In addition, our data includes information on NHS funded patients who received elective surgery from privately owned providers. However, as it has been well documented, HES data has poor coverage of care delivered in private settings. This does not present a problem for our analysis, since during our period of analysis, private providers accounted for less than 3% of total NHS volume. Approximately 5% of our sample received elective surgery in privately owned facilities. In addition, consistent with other research (Gaynor *et al*., 2010; Propper and van Reenen, 2010), and NHS data cleaning rules, we limit our analysis to providers who treat over 99 AMIs per year.

9 This generalised network was provided by the Department of Transport and is populated with road link-specific travel speeds derived from their National Transport Model for 2003. We generated the GP hospital origin-destination matrix using the Network Analysis tools from the ESRI ArcGIS software package.

10 Our mortality measure only includes deaths that occurred within the hospital. While there are 30-day all cause AMI mortality figures, they rely on linking HES data to data on deaths provided by the Office for National Statistics (ONS). Unfortunately, those data linkages were not reliable for the years in our analysis and we find that when using them, the number of deaths in the hospital are equal to the number of total deaths out of the hospital Given that during the period we investigate, length of stay for patients with an AMI only reduced by less than 2% (from 9.43 to 9.26 days), it’s unlikely our results were driven by hospitals discharging patients ‘sicker and quicker’.

11 We choose to limit our analysis to patients with a length of stay of over two days or patients who died in the first two days of their admission in order to avoid possible up-coding, whereby patients with other conditions were coded as having an AMI in order to generate larger reimbursements. Up-coding may have been prevalent during this period as the government shifted to a fixed-price, prospective reimbursement system (Street and Maynard, 2007).

12 As Davies *et al*. (2001) note, 30-day AMI quality is used for hospital rankings by healthgrades.com, the Michigan Hospital Association (where it’s aggregated with congestive heart failure and angina), the UK Department of Health, the California Hospital Outcomes Project, the Greater New York Hospital Association, and the University Hospital Consortium.

13 See http://2008ratings.cqc.org.uk/findcareservices/informationabouthealthcareservices.cfm

14 Dr Foster is a commercial company that measures provider performance in the the NHS; see http://www.drfosterhealth.co.uk, last accessed March 1, 2011.

15 For confidentiality reasons, the patient home addresses are not available for use in our analysis. However, we do have access to codes that identify the patient’s GP and GP postcode. There are around 7600–7700 GP postcodes in each year in our data. Patients can usually (at the time relevant for our study) only register at a GP practice if they live in the catchment area of that GP, so GPs serve as a strong proxy for patients’ home addresses.

16 The Charlson index of co-morbidities is a zero to six ranking of illness severity, which predicts the probability of a patients’ one-year mortality based on the presence of certain co-morbidities. It is calculated based on the presence of illnesses described in the secondary diagnosis field within the HES dataset.

17 It is important to note that GPs do not need to refer patients to a particular hospital within their market for the hospital to be included in the *nlhhi* associated with the GP.

18 Patient characteristics included age, gender, socio-economic status and severity of illness. Provider characteristics included indicator dummies for teaching status, public or private ownership and FT status. Distance was measured as the straight-line distance from GPs to hospital sites.

19 Following Kessler and McClellan (2000), we used these predicted counts (*count*_*jk*_), which is the expected number of patients each GP *j* refers to hospital *k* in a given year to generate market shares, denoted *α*_*jk*_, for all hospitals *k =* 1,*…,K,* located within 100 km of *j:*

We then used these predicted market shares generated from the predicted count of patients referred from *j* to *k* to create GP-level HHIs. These GP-level HHIs were the sum of the squares of the predicted market shares of providers within 100 km of *j:*

This measure would be similar to our fixed radius HHIs centred on GP practices, except that the market shares used to create the *HHI*_*j*_ would be based on predicted patient flows. Consistent with Kessler and McClellan (2000), we weight these GP HHIs by *β*_*jk*_, which is the share of patients from GP *j* which comprise the total activity of hospital *k,* in order to generate hospital level HHIs that are vary based on the competitiveness of the various GP markets served by hospital *k*, such that

and,

However, as Kessler and McClellan (2000) noted, since there is potential that unobserved determinants of patients’ hospital choice could be correlated with patients’ underlying health status, we assign *HHI*_*k*_ back to GP practices based on the shares of patients from GP *j* treated at all hospitals *k =* 1,…, *k* located within a 100 km radius of *j:*

This *HHI*_*j**_ is calculated for all years and for all five procedures and then averaged across the five procedures weighted by the total volume of each procedure performed per year. Thus, this measure varies both by year and by GP.

20 This is easily seen when our various concentration measures are superimposed onto a map of England. These mappings are available from the authors on request.

21 A GP is located in an urban area if the local output area falls within urban settlements with a population of 10,000 or more, according to the UK Office of National Statistics - available at http://www.statistics.gov.uk/geography/census_geog.asp.

22 Note that we limit observations in our analysis to patients who travelled for care within their relevant geographic market. For example, if we measured competition within fixed 30 km markets, we limited our observations who travelled less than 30 km for care. We introduced this limit so that if a patient had a heart attack away from home, it would not indicate that their local market structure was associated with the location where they received care. This is why the number of observations in our analysis varies according to the measure of market structure used in the particular estimation.

23 Within our HES data, the area is defined as a city if the population within the hospital’s output area is greater than 10,000. We also estimated an interaction between our time trends and a London dummy and this was also not significant.

## Appendix A: Bias Mitigation from Using Non-elective Outcomes When Competition is Measured by Elective Procedures

### Appendix

The pervasive problem in studies of the effects of elective market structure on hospital quality is that the measures of market structure are endogenous to quality in cross-sectional analysis. Our main strategy in this article for solving this problem is to use the timing of the NHS reforms interacted with measures of market structure to provide an exogenous source of variation in competition over time. We back this up with estimates based on market structure indices predicted from exogenous characteristics (the Kessler and McClellan (2000) index) and instrumental variables based on dispersion in GP-hospital distances. We also argue that using non-elective AMI procedure mortality as a quality indicator mitigates (though does not eliminate) the potential bias from the endogeneity of market structure indices to elective health care quality. Our reasoning is set out below.

The underlying parameter we would like to estimate is the causal effect of the market structure for electives *comp_e* on latent hospital quality *q*. Note, by definition, choice is only operational for electives, so it is only elective procedures that provide any quality incentives to hospitals. For simplicity, assume that quality is influenced by elective market structure and by an exogenous quality component *u*:

A1

There are possibly many potential measures of hospital quality *q* based on different procedures *j*. Shock *v_j* captures idiosyncratic procedure-specific quality differences and *v_j* is assumed uncorrelated with *q* and uncorrelated with *v_k, for j ≠ k*:

so substituting in (A.1)

An inherent endogeneity issue arises in estimating this equation for elective procedures

A2

The measured market structure *comp_e* is dependent partly on exogenous geographical factors *w,* but also partly on patient choices in response to observed elective quality e.g.:

A3

or in reduced form

where

So the bias in using elective procedures as a quality measure in (A.2) is:

Now, suppose we have an alternative measure of quality from non-elective AMI outcomes:

So what we estimate now is:

Now the bias is:

(as long as Var(*v_e*) > 0, and c > 0 in (A.3)).

Put simply, the bias in using elective procedures includes an additional bias induced by people choosing hospitals based on all dimensions of elective quality (general and idiosyncratic). For AMIs, this bias is reduced since individuals do not generally have a choice over where they receive care and the bias is only attributable to the components of AMI quality that are shared with elective quality, not to the idiosyncratic part of elective quality.

## Appendix B: Key Summary Statistics

### Appendix

**Table:**

Variable name	Variable description	Mean	Standard deviation	Minimum	Maximum
*Death_30*	Death_30 is a binary indicator that equals 1 if the patient died within 30-days of being admitted with an AMI	0.1369	0.3437	0	1
*Age*	Age is the age of the patient	71.4371	12.8655	40	99
*Female*	Female is an indicator that equals 1 if the patient’s gender is female	0.3813	0.4857	0	1
*IMD_Income_2007*	IMD_Income_2007 is score of 1–5 based on the income component of the 2007 index of multipledeprivations.	3.1607	1.3982	1	6
*Tradiditional_NHS**	Traditional_NHS is an indicator that equals 1 if the hospital where the patient received care is not a foundation trust or teaching hospital	0.7239	0.4471	0	1
*Teaching**	Teaching is an indicator variable that equals 1 if the hospital where the patient is treated is a teachinghospital	0.1458	0.3529	0	1
*FT**	FT is an indicator variable that equals 1 if the hospital where the patient is treated is a foundation trust	0.1605	0.3671	0	1
*Charlson_Score*	The Charlson comorbidity score is an index ranging from 0 to 6, based on the patient’s co-morbidities. Six is the most severe.	1.7047	1.0543	0	12
*Angioplasty*	Angioplasty is an indicator variable that equals 1 if the patient underwent an angioplasty during his/her admission	0.0543	0.2267	0	1
*Negloghhi 30 km*	Negloghhi30 km is the negative log of the HHI measured using a fixed radius that is centred on each GP practice and averaged across 2002 to 2005	1.4768	0.9186	0	3.6085
*Negloghhi 30 band*	Negloghhi30band is the negative log of the HHI measured using a variable radius that is defined by the 30-min travel time surrounding each GP practice and averaged across 2002 to 2005	1.2526	0.8141	0	3.2266
*Negloghhi 95*	Negloghhi95 is the negative log of the HHI measured using a variable radius that captures the 95th percentile of travel for each GP practice	0.7349	0.5561	0	3.728348
*Neglohhhi 20*	Negloghhhi20 is the negative log of the HHI measured using a fixed radius of 20 km drawn around each hospital and averaged across 2002 to 2005	1.0069	0.9292	0	3.3369
*Log G_Count_30mins*	Log G_Count_30Band is the logged count of hospitals within a variable radius that captures a 30-min travel time surrounding each GP practice and averaged across 2002 to 2005	1.5651	0.8600	0.0000	3.4657
*Log G_Count_30km*	Log G_Count_30 km is the logged count of hospitals within a fixed radius that extends 30 km around each GP practice	1.7806	0.9723	0.0000	3.8501
*Log G_Count_95sh*	Log G_Count_95 is the logged count of the hospitals within a radius defined by the distance that captures the 95th percentile of travel for each GP practice and averaged across 2002 to 2005	1.3985	0.7113	0.0000	3.7992
*Distance*	Distance is the straight-line distance that each patient travelled for care measured in kilometres	13.3152	28.4493	0.0000	607.5968
*Site Activity*	The number of heart attack patients each hospital treats annually.	483.9835	233.7503	100	1,312

* The cumulative sum of Traditional NHS, FT and Teaching is greater than 1.00 because hospitals can be both teaching hospitals and foundation trusts. Observations are limited to patients between 39 and 100 years of age with a length of stay greater than two days, treated at sites that treated more than 25 AMIs per year. Unlike the regressions that we present, we do not limit the distance that patients travelled for care. AMI, acute myocardial infarction; NHS, National Health Service; FT, foundation trust; HHI, Herfindahl-Hirschman Indexes.

## Appendix C: Least Squared Estimates of (1) with Competition Measured as the Negative ln of the HHI Within a Market That Captures All Hospitals Within the 95th Percentile of each GP’s Maximum Travel Distance

### Appendix

**Table:**

	Coefficient	Standard error
*2002–2005 Trend*	−0.0024***	0.0002
*2006–2008 Trend*	0.0014**	0.0004
*2002–2005 Trend ×* *nlhhi*	0.0002	0.0002
*2006–2008 Trend ×* *nlhhi*	−0.0014**	0.0005
*Negloghhi95*	−0.0014	0.0028
*Female*	0.0124***	0.0012
*Charlson2*	0.0361***	0.0013
*Charlson3*	0.0776***	0.0021
*Charlson4*	0.1242***	0.0034
*Charlson5*	0.1399***	0.0055
*Charlson6*	0.1928***	0.0071
*IMD Income 2*	0.0011	0.0018
*IMD Income 3*	0.0055*	0.0018
*IMD Income 4*	0.0046*	0.0019
*IMD Income 5*	0.0059*	0.0021
*Age 45–49*	−0.0016	0.0022
*Age 50–54*	0.0053*	0.0021
*Age 55–59*	0.0125***	0.0021
*Age 60–64*	0.0263***	0.0021
*Age 65–69*	0.0446***	0.0022
*Age 70–75*	0.0741***	0.0023
*Age 75–79*	0.1157***	0.0023
*Age 80–84*	0.1539***	0.0024
*Age 85–89*	0.1974***	0.0027
*Age 90+*	0.2564***	0.0035
*Teaching*	−0.0056	0.0123
*FT*	0.0050*	0.0022
*Site Activity (150–300)*	−0.005	0.0037
*Site Activity (300–450)*	−0.0180***	0.0039
*Site Activity (450 + )*	−0.0254***	0.0042
*Distance*	0.0008***	0.0002
*Angioplasty*	−0.0460***	0.0023
*February*	−0.0042	0.0026
*March*	−0.0093***	0.0025
*April*	−0.0060*	0.0025
*May*	−0.0100***	0.0025
*June*	−0.0120*	0.0025
*July*	−0.0110***	0.0025
*August*	−0.0103***	0.0026
*September*	−0.0109***	0.0026
*October*	−0.0068**	0.0025
*November*	−0.0044	0.0026
*December*	−0.0025	0.0025
*Tuesday*	−0.0062***	0.0018
*Wednesday*	0.0000	0.0018
*Thursday*	−0.0027	0.0018
*Friday*	−0.0234***	0.0017
*Saturday*	0.0910***	0.0025
*Sunday*	0.2400***	0.0035
*North East × Year*	−0.0003	0.0001
*Yorkshire and Humber × Year*	−0.0001	0.0001
*North West × Year*	0.0001*	0.0000
*East Midlands × Year*	0.0000	0.0000
*West Midlands × Year*	0.0000	0.0000
*East of England × Year*	0.0000	0.0001
*South East Coast × Year*	0.0000	0.0000
*South Central × Year*	−0.0001	0.0001
*South West × Year*	0.0000	0.0000
*Hospital Fixed Effects*	Yes	
*GP Fixed Effects*	Yes	
*N*	422,350	
R^2^	0.126	

**Notes:** * Significant at 10% level. ** Significant at 5%. *** Significant at 1%. Dependent variable = 1 if patient died within 30-days of their admission to hospital. Standard errors are clustered on GP-practices. Reference categories: Male, Charlson1, IMD-Income1, Age 40–44, Traditional NHS Trust, Site Activity (0–150), January, London SHA. FT, foundation trust; HHI, Herfindahl-Hirschman Indexes; SHA, Strategic Health Authorities.

## Appendix D: First Stage of IV Estimate – Dependent Variable: 2002–2005 × nlhhi

### Appendix

**Table:**

Variable	Coefficient	Standard error
*2002–2005 Trend*	1.0261***	0.0153
*2006–2008 Trend*	0.4037***	0.0222
*Female*	−0.0063	0.0183
*Charlson 2*	−0.0061	0.0197
*Charlson 3*	−0.0300	0.0295
*Charlson 4*	−0.0376	0.0436
*Charlson 5*	0.0223	0.0734
*Charlson 6*	−0.1546	0.0878
*IMD Income Vector – 2*	−0.0018	0.0302
*IMD Income Vector – 3*	−0.0029	0.0309
*IMD Income Vector – 4*	−0.0044	0.0318
*IMD Income Vector – 5*	−0.0374	0.0339
*Age 45–49*	−0.0564	0.0725
*Age 50–54*	−0.0259*	0.0676
*Age 55–59*	−0.1098	0.0646
*Age 60–64*	−0.0393	0.0628
*Age 65–69*	−0.0669	0.0617
*Age 70–74*	−0.0172	0.0629
*Age 75–79*	−0.0222	0.0620
*Age 80–84*	−0.0135	0.0615
*Age 85–89*	−0.0046	0.0640
*Age 90 +*	−0.0487	0.0681
*Teaching Hospital*	−0.6379	0.3280
*Foundation Trust*	−1.7376***	0.1669
*Angioplasty*	−0.0564	0.0729
*Hospital Volume (150 AMIs – 299)*	−0.0381	0.1715
*Hospital Volume (300 AMIs – 449)*	−0.2220	0.1899
*Hospital Volume (450 AMIs +)*	0.5298*	0.2155
*Distance to Nearest Provider*	−0.0002	0.0001
*Distance to Second Nearest Provider*	0.0000	0.0000
*Distance to Third Nearest Provider*	0.0000	0.0000
*Distance to Fourth Nearest Provider*	−0.0001***	0.0000
*February*	−0.0533	0.0366
*March*	−0.0574	0.0356
*April*	−0.0607	0.0375
*May*	−0.0900*	0.0370
*June*	−0.0003	0.0370
*July*	−0.1184*	0.0391
*August*	−0.0663	0.0398
*September*	−0.1409***	0.0396
*October*	−0.2610***	0.0419
*November*	−0.2006***	0.0413
*December*	−0.2095***	0.0406
*North East × Year*	−0.0180	0.0125
*Yorkshire and Humber × Year*	−0.0085	0.0066
*North West × Year*	0.0062	0.0042
*East Midlands × Year*	−0.0028	0.0003
*West Midlands × Year*	0.0018	0.0006
*East of England × Year*	−0.0004	0.0010
*South East Cost × Year*	0.0032	0.0004
*South Central × Year*	−0.0042	0.0006
*South West × Year*	−0.0039	0.0046
*Tuesday*	−0.0478	0.0273
*Wednesday*	−0.0637	0.0284
*Thursday*	0.0076	0.0269
*Friday*	−0.0181	0.0266
*Saturday*	−0.0034	0.0332
*Sunday*	−0.0369	0.0414
*2002–2005 Trend*	0.0375***	0.0025
*2006–2008 Trend*	1.0843***	0.0120
*Female*	0.0048	0.0070
*Charlson 2*	−0.0035	0.0073
*Charlson 3*	−0.0094	0.0111
*Charlson 4*	−0.0176	0.0166
*Charlson 5*	0.0084	0.0280
*Charlson 6*	−0.0299	0.0344
*IMD Income Vector – 2*	−0.0004	0.0114
*IMD Income Vector – 3*	0.0041	0.0116
*IMD Income Vector – 4*	0.0110	0.0118
*IMD Income Vector – 5*	−0.0062	0.0123
*Age 45–49*	−0.0040	0.0267
*Age 50–54*	−0.0020	0.0249
*Age 55–59*	−0.0345	0.0236
*Age 60–64*	−0.0059	0.0231
*Age 65–69*	−0.0076	0.0227
*Age 70–74*	0.0103	0.0229
*Age 75–79*	0.0015	0.0229
*Age 80–84*	0.0058	0.0224
*Age 85–89*	0.0124	0.0238
*Age 90 +*	−0.0091	0.0258
*Teaching Hospital*	−0.7666***	0.1115
*Foundation Trust*	−0.6250***	0.0626
*Angioplasty*	−0.0998**	0.0299
*Hospital Volume (150 AMIs – 299)*	−0.3386***	0.0681
*Hospital Volume (300 AMIs – 449)*	−0.3902***	0.0744
*Hospital Volume (450 AMIs +)*	−0.0083	0.0821
*Distance to Nearest Provider*	−0.0001	0.0000
*Distance to Second Nearest Provider*	0.0000	0.0000
*Distance to Third Nearest Provider*	0.0000	0.0000
*Distance to Fourth Nearest Provider*	0.0000***	0.0000
*February*	0.0032	0.0120
*March*	−0.0085	0.0117
*April*	0.0305*	0.0127
*May*	0.0095	0.0128
*June*	0.0275*	0.0126
*July*	0.0317	0.0140
*August*	0.0470**	0.0141
*September*	0.0246	0.0140
*October*	0.0287	0.0154
*November*	0.0560***	0.0150
*December*	0.0587***	0.0145
*North East × Year*	−0.0089	0.0054
*Yorkshire and Humber × Year*	−0.0048	0.0038
*North West × Year*	0.0018	0.0013
*East Midlands × Year*	−0.0013	0.0001
*West Midlands × Year*	0.0012	0.0003
*East of England × Year*	0.0001	0.0003
*South East Cost × Year*	0.0015	0.0002
*South Central × Year*	−0.0010	0.0002
*South West × Year*	−0.0030	0.0030
*Tuesday*	−0.0133	0.0106
*Wednesday*	−0.0184	0.0107
*Thursday*	0.0003	0.0103
*Friday*	−0.0014	0.0101
*Saturday*	0.0125	0.0128
*Sunday*	−0.0121	0.0154
*2002–2005 Trend*	−0.0023**	0.0007
*2006–2008 Trend*	0.0173***	0.0012
*Female*	−0.0018	0.0010
*Charlson 2*	−0.0020	0.0011
*Charlson 3*	−0.0043*	0.0016
*Charlson 4*	−0.0036	0.0023
*Charlson 5*	0.0022	0.0039
*Charlson 6*	−0.0104*	0.0045
*IMD Income Vector – 2*	−0.0006	0.0016
*IMD Income Vector – 3*	0.0000	0.0017
*IMD Income Vector – 4*	−0.0025	0.0017
*IMD Income Vector – 5*	−0.0028	0.0019
*Age 45–49*	−0.0053	0.0040
*Age 50–54*	−0.0022	0.0038
*Age 55–59*	−0.0027	0.0037
*Age 60–64*	−0.0012	0.0035
*Age 65–69*	−0.0044	0.0035
*Age 70–74*	−0.0019	0.0035
*Age 75–79*	−0.0034	0.0035
*Age 80–84*	−0.0030	0.0035
*Age 85–89*	−0.0026	0.0035
*Age 90 +*	−0.0044	0.0038
*Teaching Hospital*	0.0176	0.0252
*Foundation Trust*	0.0388*	0.0070
*Angioplasty*	0.0068	0.0033
*Hospital Volume (150 AMIs – 299)*	0.0018	0.0092
*Hospital Volume (300 AMIs – 449)*	0.0155	0.0103
*Hospital Volume (450 AMIs +)*	0.0126	0.0115
*Distance to Nearest Provider*	0.0000*	0.0000
*Distance to Second Nearest Provider*	0.0000***	0.0000
*Distance to Third Nearest Provider*	0.0000	0.0000
*Distance to Fourth Nearest Provider*	0.0000***	0.0000
*February*	−0.0036	0.0021
*March*	−0.0031	0.0021
*April*	−0.0067	0.0022
*May*	−0.0071	0.0021
*June*	−0.0005	0.0021
*July*	−0.0095***	0.0022
*August*	−0.0104***	0.0022
*September*	−0.0127***	0.0023
*October*	−0.0164***	0.0023
*November*	−0.0145***	0.0023
*December*	−0.0135***	0.0023
*North East × Year*	0.0001	0.0004
*Yorkshire and Humber × Year*	0.0003	0.0003
*North West × Year*	0.0005	0.0003
*East Midlands × Year*	0.0000	0.0000
*West Midlands × Year*	0.0000	0.0000
*East of England × Year*	−0.0001	0.0001
*South East Cost × Year*	0.0001***	0.0000
*South Central × Year*	0.0000	0.0000
*South West × Year*	0.0004	0.0004
*Tuesday*	0.0012	0.0015
*Wednesday*	0.0011	0.0016
*Thursday*	0.0015	0.0015
*Friday*	0.0000	0.0015
*Saturday*	−0.0006	0.0018
*Sunday*	−0.0007	0.0023

**Notes:** * Significant at 5% level. ** Significant at 1%. *** Significant at 0.1%. Error terms are clustered on GP practices.
